# Spontaneous Intraoperative Rupture of a Large Interstitial Pregnancy: Laparoscopic Management

**DOI:** 10.1155/2020/5626783

**Published:** 2020-04-14

**Authors:** Ourania Koukoura, George Dragoumis, Georgia Gorila, Irontianta Gkorezi-Ntavela, Konstantinos Dafopoulos, George Pistofidis

**Affiliations:** ^1^Obstetrics and Gynecology Department, University Hospital of Larissa, Thessaly, Greece; ^2^Tertiary Referral Center of Gynecological Laparoscopy, Lefkos Stavros Hospital, Athens, Greece

## Abstract

We present a case of a large interstitial pregnancy which was intraoperatively ruptured, but was eventually laparoscopically treated. The patient experienced 9 weeks of amenorrhea, and a right cornual pregnancy measuring 6 cm was diagnosed. The patient consented on having a minimal surgical treatment, and a laparoscopic right cornuotomy was decided. During surgery, and prior to any manipulation to the uterus, there was a spontaneous rupture of the ectopic which resulted in excessive bleeding. Temporal pressure at the bleeding site and ligation of the superior branches of the right uterine artery allowed for a careful dissection of the right uterine cornua and achieved hemostasis. The surgery proceeded uneventfully thereafter. Although surgical intervention in such cases entails a high risk of hemorrhage, successful completion of the laparoscopy lies on the meticulous preoperative planning and the controlled precise surgical steps during the procedure.

## 1. Background

Interstitial pregnancy is a rare event constituting 2-4% of all tubal ectopic pregnancies [1, 2]. It is defined as the ectopic gestation developing in the uterine part of the fallopian tube (a thick section averaging 0.7 mm in diameter and 1–2 cm in length) and is characterized by a significantly greater propensity to expand before rupture as compared with the distal portion [1, 3]. For these reasons, it may remain asymptomatic until 7–16 weeks of gestation [4–6]. It is associated with maternal mortality and morbidity due to hemorrhage. Interstitial pregnancy is managed medically with methotrexate or surgically with laparoscopy or laparotomy (cornual wedge resection or hysterectomy). Laparoscopic treatment may be considered the gold standard nowadays and has several advantages over laparotomy. These include decreased surgical morbidity, lower healthcare cost, shorter hospital stay, and faster return to normal activities [6, 7]. It is mandatory though that laparoscopic surgery be conducted by experienced surgeons so that potential adverse intraoperative events can be managed appropriately. We present a case of a large interstitial pregnancy which was treated laparoscopically. Intraoperative rupture of the ectopic resulted in severe bleeding which was successfully managed.

## 2. Case Report

**Figure 1:**
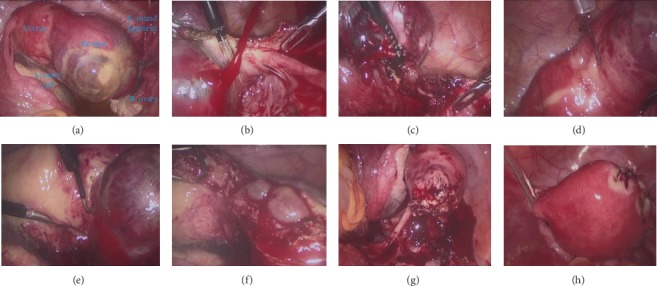
(a) The anatomical relation with other pelvic structures is shown in this panel. (b) Profuse bleeding caused by spontaneous rupture of the ectopic. (c) The superior branches of the right uterine artery were coagulated and transected. (d) Injection of diluted vasopressin in the myometrium, close to the border of the ectopic. (e) The uterine wall is incised with monopolar cautery. (f) The gestational sac is exposed and ruptured during dissection. (g) A portion of the uterine horn has been excised. (h) The uterine wound was closed with interrupted sutures.

A 39-year-old woman (gravida 1, para 0) was referred to our hospital for a suspected ectopic pregnancy. She presented with right iliac fossa pain and 9 weeks of amenorrhea. Medical history was negative regarding previous pelvic or genital infections or abdominopelvic surgeries. Her serum *β*-HCG was 18900 mIU/mL, and a transvaginal scan revealed an empty uterus and a hypoechoic mass of 5.7 cm in the right adnexa with no visible fetus. Patient consent was obtained for laparoscopic excision of the ectopic. The laparoscope was inserted into the abdominal cavity from an 11 mm supraumbilical incision using an open access technique. Three additional trocars were introduced under direct vision. Based on visual inspection of the pelvic cavity, an interstitial pregnancy was identified in the right uterine horn close to the round ligament. The diameter of the ectopic pregnancy exceeded the diameter of the uterus (Figure 1(a)). During incision of the round ligament, spontaneous rupture of the ectopic sac occurred, resulted in profuse bleeding (Figure 1(b)). Exertion applied on the ectopic sac seemed to somewhat control the bleeding. Pressure on the ectopic allowed better visualization of the broad ligament which was then coagulated and incised. The superior branches of the right uterine artery were then coagulated and transected. (Figure 1(c)) Bleeding was thus reasonably controlled. Diluted vasopressin was injected (1 mL of 20 U of vasopressin diluted with 40 mL of normal saline) in the myometrium close to the border of the ectopic (Figure 1(d)). The uterine wall was then incised with monopolar current, set at 30 W (Figure 1(e)). During dissection, the gestational sac was exposed and ruptured (Figure 1(f)). The whole of the ectopic was removed with a portion of the uterine horn (Figure 1(g)). A laparoscopic bag was used to remove the ectopic from the abdominal cavity. Interrupted inverted sutures (Monocryl No. 1) were placed at the uterine wound (Figure 1(h)). Adequate hemostasis was achieved. A drain was placed in the pouch of Douglas and left for 24 hours. The operation lasted approximately 160 minutes, and the estimated blood loss was 800 mL. No transfusion was necessary. Postoperative course was uneventful, and the patient was discharged two days later. The *β*-HCG levels were below 5 IU/mL, three weeks after the operation.

## 3. Discussion

Interstitial ectopic pregnancy is implanted in the interstitial portion of the fallopian tube that originates at the tubal ostium and traverses into the muscular wall of the uterus. Interstitial pregnancy is frequently referred as a “cornual pregnancy.” The diagnosis of cornual pregnancy, however, should only be used when the pregnancy is located within a noncommunicating horn of a bicornuate uterus [8]. Traditionally, interstitial pregnancy was managed with either methotrexate or laparotomy with cornual wedge resection or hysterectomy. Nowadays, laparoscopy is becoming the treatment of choice for an increasing number of cases.

Medical treatment may be considered in cases of early, asymptomatic interstitial pregnancy when *β*-HCG is less than 4,000 IU/L, ectopic size less than 4 cm, and/or when the exact location of the pregnancy is uncertain. To minimize the risk of conservative treatment failure, surgical treatment should be performed in cases of interstitial pregnancy sized more than 4 cm and/or in cases where there is visible fetal heart rate [5]. Both criteria were met in our case since *β*-HCG was 18,900 IU/mL and the size of the ectopic was 6-7 cm. In most reported cases, the size of the ectopic does not exceed that of 4.5 cm with an average diameter of 2.5-3.5 cm [7, 9].

Interstitial pregnancies can remain asymptomatic until late in gestation before a sudden massive hemorrhage. The risk of bleeding is increased due to the vascular anastomoses of ovarian and uterine vessels in this region [2]. Accurate diagnosis of an interstitial pregnancy lies primarily on the timing of the transvaginal scan. Early in the first trimester, several criteria can be identified that will guide the diagnosis. An empty uterine cavity, a chorionic sac at least 1 cm from the lateral edge of the uterine cavity, and a thin (<5 mm) myometrial layer on the border of the gestational sac have all been outlined as the main sonographic features for diagnosis [10]. In many cases, however, definite diagnosis can be made only during surgery. Early detection of an interstitial pregnancy provides the opportunity for medical management. Advantages of medical treatment include avoidance of a surgical scar on the uterus and other associated risks of surgery. Nevertheless, methotrexate treatment success rate is 80% as reported in many case series [11]. A possible treatment failure can lead to multiple dosages, subsequent cornual rupture, and life-threatening hemorrhage. The most important prognostic factor for methotrexate success is the initial *β*-HCG level. Ultrasound-guided local injection of methotrexate has been successfully used in cases where conservative treatment is acceptable [11, 12].

As laparoscopic techniques improved, laparoscopy became the “gold standard,” and most of the cases were managed laparoscopically. Good laparoscopic technique ensures the reduced blood loss, reduced risk of thromboembolism, less postoperative pain, and shorter hospital stay [13]. We performed cornual resection of the uterus following ligation of the superior branches of the uterine artery. Injection of diluted vasopressin facilitated control of bleeding during myometrial resection. A less invasive surgical approach has been proposed in similar cases, i.e., cornuostomy, where the pregnancy is removed without removing any of the surrounding myometrium [14]. Although cornuostomy and cornual resection have comparable outcome, it is postulated that cornuostomy is associated with shorter operation time and less anatomical damage to the uterus. Our patient consented and ended up having a cornual resection of the uterus; however, in retrospect, we wonder whether a cornuostomy was feasible in our case. We trust that the intraoperative spontaneous rupture of the ectopic which led to severe bleeding guided towards a more radical surgical approach.

Devascularization of the uterus together with vasoconstrictive agents made the operation safe and the outcome favorable. Intramyometrial injection of diluted vasopressin is frequently used in similar cases. Although laparoscopic cornual resection is a safe and definite treatment, it requires good laparoscopic technique and high awareness of the possible intraoperative complications, to achieve excellent outcome and uneventful postoperative recovery.
